# Psychosocial Issues Related to Newborn Screening: A Systematic Review and Synthesis

**DOI:** 10.3390/ijns8040053

**Published:** 2022-09-27

**Authors:** Audrey Tluczek, Anne L. Ersig, Shinhyo Lee

**Affiliations:** 1 School of Nursing, University of Wisconsin-Madison, 701 Highland Ave, Madison, WI 53705, USA; 2 School of Nursing, Columbia University, 560 W 168th St, New York, NY 10032, USA

**Keywords:** newborn screening, psychosocial

## Abstract

Genomic advances have contributed to a proliferation of newborn screening (NBS) programs. Psychosocial consequences of NBS have been identified as risks to these public health initiatives. Following PRISMA guidelines, this systematic review synthesizes findings from 92 evidence-based, peer-reviewed research reports published from 2000 through 2020 regarding psychosocial issues associated with NBS. Results describe parents’ knowledge of and attitudes towards NBS, reactions to and understanding of positive NBS results, experiences of communication with health providers, decisions about carrier testing, and future pregnancies. Findings also explain the impact of positive NBS results on parent–child relationships, child development, informing children about carrier status, family burden, quality of life, and disparities. In conclusion, psychosocial consequences of receiving unexpected neonatal screening results and unsolicited genetic information remain significant risks to expansion of NBS. Findings suggest that risks may be mitigated by improved parent NBS education, effective communication, individualized genetic counseling, and anticipatory developmental guidance. Clinicians need to take extra measures to ensure equitable service delivery to marginalized subpopulations. Future investigations should be more inclusive of culturally and socioeconomically diverse families and conducted in low-resource countries. Providing these countries with adequate resources to develop NBS programs is an essential step towards achieving international health equity.

## 1. Introduction

Newborn screening (NBS) programs are public health initiatives that screen infants shortly after birth to identify those at risk for serious health conditions, most of which are genetic. Early diagnosis and treatment can reduce infant mortality, morbidity, and long-term complications [1]. In 1962, the United States (US) implemented a mass NBS program for phenylketonuria (PKU) [2], marking a paradigm shift from diagnosis based on clinical presentation to identifying pre-symptomatic infants. In 1968, the World Health Organization (WHO) established criteria requiring conditions on NBS panels to have established treatments [3]. In 2004, the American College of Medical Genetics surveyed experts and stakeholders to develop new NBS criteria, and recommended a core panel of 29 conditions for all states [4]. The new criteria included conditions for which early detection offered potential long-term benefits to children and families. The WHO criteria were expanded in 2008 to include less well-known conditions, which created opportunities to document the natural clinical course of such conditions, produced more precise diagnostic tests, and led to more effective treatments [5].

Countries worldwide have NBS programs that screen for a variety of conditions. Most panels include core conditions and secondary conditions [6]. Core conditions are those for which there is “compelling evidence of benefit” for early detection, intervention, and treatment. Secondary conditions are identified incidentally, and have less evidence of benefit from early detection [7,8].

Since its inception, NBS has generated debate. Proponents point to the benefits of early treatment to improve the health and lifespans of affected children, while families avoid a costly, stressful, and protracted diagnostic process. Preventing diagnostic delays in underrepresented or economically underserved populations also advances health equity [9]. Identifying more complex inherited disorders through tandem mass spectrometry and other novel genetic technologies further expands knowledge of risk beyond single-gene conditions [10,11]. Future use of whole genome sequencing in NBS could exponentially increase the number of conditions identified, benefiting ever-increasing numbers of children.

Conversely, concerns about the psychosocial ramifications of NBS for parents and infants with abnormal (hereafter, “positive”) results remain. Tandem mass spectrometry detects mild conditions that require no treatment. Advanced technologies identify unaffected heterozygote carriers, for whom NBS may provide no known health benefits. Findings can also reveal non-paternity. Parents’ lack of knowledge about NBS and genetics lead to confusion about results and the need for subsequent diagnostic testing—all of which can engender parental distress and worry about the infant’s health [1,12].

A systematic review of 28 studies published between 1981–2000 found that positive results were associated with parental emotional distress due to poor provider communication, particularly for false-positive results [12]. In many European countries, parental consent is required [13]. In other countries, such as the US, consent is not required and NBS is mandatory except for religious exemptions. Thus, results come as a shock to parents during a vulnerable time in their lives when they are likely to feel fatigued and overwhelmed and may be struggling with postpartum depression [14]. Unsolicited genetic information from NBS can also impact parents’ future reproductive decisions, raising concerns about state interference with private family matters [15]. While experts recognize the importance of understanding the psychosocial consequences of imparting unexpected genetic risk information to parents, and the effects on their relationships with their child [16], no global reviews of such issues within the context of NBS have been completed since the expansion of NBS in the early 2000s. The purpose of this systematic literature review was to synthesize the most recent empirical evidence regarding psychosocial issues associated with NBS in the era of genetic technologies.

## 2. Methods

A team of content and methods experts followed PRISMA guidelines in conducting this systematic review of peer-reviewed research publications that focused on psychosocial issues associated with NBS [17]. For this project, psychosocial was defined as the “interaction of social, cultural, and environmental influences on the mind and behavior” [18], and also included factors related to human emotion, cognition, child development, and interpersonal relationships [19].

We included qualitative, quantitative, and mixed methods studies in which participants were parents of any age with children of any age who underwent NBS. We also included third party observations of families affected by positive NBS results. All articles were written in English and published between 2000 and 2020 in peer-reviewed journals. We excluded systematic or narrative reviews, opinion papers, study proposals, secondary sources, dissertations, and parent reports about hypothetical situations or opinions about NBS if they did not have a child who underwent NBS. See Table 1 for terms and databases used for this literature review.

**Figure 1:**
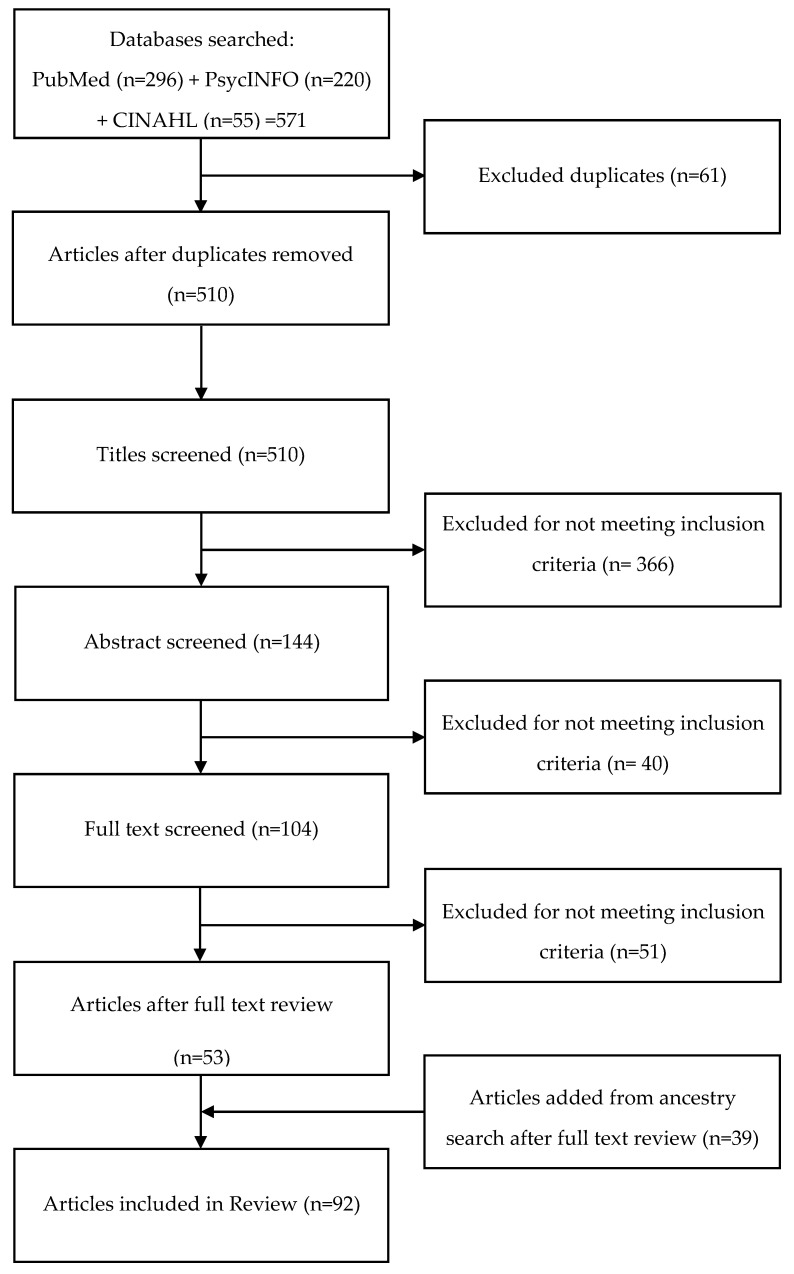
Screening Flow Chart.

Using an eligibility checklist, two team members screened each article first by title, then abstract, and finally full text, sorting each article into three categories: *yes* (include), *maybe* (might include), and *no* (exclude) [20]. Discrepancies were addressed during team meetings. We repeated these procedures with an ancestry search of references from included articles. Figure 1 illustrates the screening and selection process.

### Data Extraction, Analysis and Synthesis

Data extracted from each article were stored in separate Word documents. To ensure accuracy and reliability, two team members extracted data independently. Regular team meetings were held to discuss and resolve inconsistencies and confirm accuracy of extracted data. Concurrent with data extraction, we conducted thematic analyses to inductively identify categories and manifest patterns in summary data [21] and incorporated sub-themes to describe specific issues. Finally, we noted gaps in the empirical evidence and directions for future NBS research.

## 3. Results

### 3.1. Search and Screening Results

The initial search of PubMed, PyscINFO, and CINAHL produced 571 articles, with 511 remaining after duplicates were removed (Figure 1, Table 2). The screening process reduced the number of included articles to 53. An ancestry search of reference lists from these articles produced an additional 39 articles. Thus, the final review included 92 articles listed in Table 3.

Studies were conducted in 12 countries, though the majority took place in North America (59.8%, *n* = 55) and Europe (26.1%, *n* = 24). Conditions screened included cystic fibrosis (CF, *n* = 53), sickle cell anemia and other hemoglobinopathies (SCD, *n* = 13), metabolic disorders (*n* = 13), type 1 diabetes (T1D, *n* = 9), congenital hypothyroidism (CH, *n* = 6), fragile X syndrome (FXS, *n* = 3), Duchenne and Becker muscular dystrophy (DMD/BMD, *n* = 2), and NBS in general (*n* = 10). Most studies used cross-sectional designs (87%, *n* = 80) and quantitative data (42%, *n* = 39), with fewer using qualitative (30%, *n* = 28) or mixed methods approaches (27%, *n* = 25). A few studies involved video observations (7%, *n* = 6). More than half of included articles focused on infants with false-positive NBS results.

### 3.2. Parent Knowledge of and Attitudes about NBS and/or Dried Blood Spots

Research repeatedly showed that parents were uninformed about NBS, conditions screened, implications of positive results, or the storage of dried blood spots (DBS) for future research [34,48,49,66,70,74]. In one study, most postpartum women with healthy infants [74] reported that they had not received prenatal education about NBS, did not know how they would receive results, and did not know that abnormal results had implications for parents. About half were unsure or did not know where to take their baby for follow-up testing if required and many did not understand that NBS identifies serious genetic disorders in infants. Postnatal education on NBS, provided by postpartum nurses or midwives, was common [23,74,75,92]. Short postpartum hospital stays, mothers’ physiological needs, and time constraints of caring for their newborns posed challenges to effective postnatal parent NBS education [74,75]. Recall improved when parents received a pamphlet about NBS postpartum, vs. prenatally [49], though findings could reflect a recency effect. By contrast, another study showed that parents preferred receiving NBS information prenatally [39].

To address gaps in parent knowledge, researchers recommended that knowledgeable health professionals provide written and verbal information [23,92], offer more information on DBS [38], and accommodate parent sociodemographics, such as income, primary language, and education [23,29,31]. Parents expressed an interest in receiving more information about the reasons for and benefits of NBS, conditions identified, how to interpret positive results, the probability of positive results, timing of receiving results, and the possibility of additional testing [92]. Expanding responsibility for family education about NBS to include primary care and prenatal providers [23,39], and improving parents’ understanding of false-positive and true-positive results, were also proposed [23]. Relying on printed material and referring parents to online resources were identified as ineffective approaches to parental education [49].

Despite the anxiety and distress associated with their experiences of NBS, parents of children with false-positive results [41,79,80,104] and children diagnosed with a condition [33,37,38] supported routine NBS. Reasons included satisfaction with communication and gratitude for receiving information about their child’s health [35,39,40] or their own carrier status [70]. Parents believed that NBS was in children’s best interest because they could benefit from early treatment [76]. Additional benefits were avoiding the emotional and financial costs of diagnostic delays, receiving information relevant to future reproductive decisions, and opportunity to prepare for having a child with special health needs [78]. However, they also highlighted the importance of parental choice [78].

Parent support for NBS programs was not universal. Mothers whose newborns were screened for FXS through a research study did so because it posed minimal risk, provided information about their child’s health and development, could inform future reproductive decisions, and was offered at no cost [88]. Reasons for declining included not wanting to know or worry, concerns about genetic testing of children, no family history of FXS, partner disapproval, infant’s apparent good health, and no cure for condition [88]. Findings point to a need for NBS protocols that guide information dissemination about essential follow-up assessments, potential treatments, ongoing family support, and communicating NBS results to all health care providers involved in the child’s care [50,78].

Parents of infants screened for genetic risk of developing T1D using next generation sequencing were less enthusiastic about expanding NBS than parents in the general population [61]. Parents voiced concerns about difficulty understanding genetic information, unclear rationale for testing at birth rather than later in childhood, limited predictive value, whether behavioral changes could mitigate risk, increased parental worry or overprotectiveness, and potential adverse effects on family functioning. Yet, parents also recognized the benefits of potentially preventing disease, particularly for conditions for which risk can be minimized [61]. Variability in parents’ support for NBS highlights the need to explore ways to help them make informed choices for their families.

Retention of dried blood spot (DBS) samples for future research raised unique concerns. Parents asserted their desire to be involved in making decisions about retaining DBS [74]. In another study, 95% of new mothers supported use of DBS for medical research, contingent upon parental informed consent and de-identification of samples [38].

### 3.3. Parent Reactions to NBS Results

#### 3.3.1. Positive (Initial) Results

Parents of infants whose NBS results were positive for SCD [22], CF [14,53,68,72,86,106], FXS [24], metabolic disorders [41,52], CH [86,92], or increased risk of T1D [16,61] reported short-term anxiety and/or stress. One study found that identifying increased genetic risk of T1D was not associated with elevated maternal anxiety [26], and another found elevated depressive symptoms in mothers with a history of postpartum depression [56]. Delays for confirmatory diagnostic testing were especially troubling for parents, some of whom reported clinical levels of depression while waiting for diagnostic sweat test results [14]. In other studies, parents reported substantial emotional distress, such as fear, shock, and worry, while waiting for results of diagnostic testing [36,51,56]. Parents whose newborns needed additional testing (e.g., a second sweat test) experienced continued distress [81], often anticipating a CF diagnosis [79]. Parents also varied in their response to uncertainty during diagnostic sweat tests [42]. Some tried to reduce uncertainty by seeking information while others maintained uncertainty by avoiding new information. Both strategies aimed to reduce or manage anxiety about a potential CF diagnosis.

In response, researchers identified ways to mitigate initial parental distress. Some occurred naturally within families. For example, spousal support was associated with less depression and stress among mothers identified as FXS carriers [24]. Interventions by health care providers included developing culturally sensitive counseling techniques [14,41], tailoring the timing, content, and method of communication to match each family’s needs for emotional support and information [14], and having knowledgeable, trained professionals communicate test results [14,53]. For CF, recommendations also included minimizing the time between receipt of NBS results and confirmatory sweat testing [14,39,47].

#### 3.3.2. True-Positive Results, Confirmed Diagnosis

A confirmed diagnosis following positive NBS results was devastating to parents who thought they had a healthy infant. The diagnosis generated a mix of shock, fear, anxiety, and disbelief [42,58,59]. Mothers of infants diagnosed with CF were more likely to have clinical levels of anxiety and depression than mothers of infants diagnosed with CH, identified as CF carriers, or with normal NBS results [99]. Delays in receiving additional information and follow-up were particularly anxiety-provoking, especially for parents who had to wait over a weekend. This prompted many to search for information from potentially unreliable online sources [57]. Parents of infants with CF, in particular, were overwhelmed by the implications of the diagnosis and the need to engage with a large and diverse health care team [57]. Grob [51] found that parents of infants diagnosed with CF through NBS felt emotionally unprepared due to lack of knowledge. They were challenged by the complexity of CF genetics and physiology and overwhelmed by the home care required to keep their child healthy, though these feelings diminished over time. The diagnosis also changed parents’ perceptions of their child from “healthy and normal” to being a child in need of additional medical care. Other responses differed, often by condition. For example, despite experiencing initial emotional distress, parents of infants with FXS viewed early diagnosis as an opportunity to prepare for their child’s future needs and to make informed reproductive plans [33].

#### 3.3.3. False-Positive Results

False-positive NBS results were particularly confusing for parents. For some conditions, such as CF and SCD, false-positive results often identified infants who are heterozygote carriers, which was difficult for parents to understand [22,95]. Many did not realize that one or both parents could also be carriers, nor did they know about carrier testing to determine risks to future pregnancies [68]. Parents of infants with positive NBS for metabolic disorders had difficulty understanding the need for additional confirmatory testing [52]. Many perceived their children as being vulnerable to health problems, and, as a result, children with false-positive results were more likely to be hospitalized or have more health care encounters than infants with normal results [52,54,59,101,107]. However, another study found that after adjusting for differences in sample demographics, there were no significant differences in health care utilization between children with false-positive CF results and those with normal results [69]. Providers’ understanding of false-positive results may have contributed to health care overutilization. Those who were less familiar with conditions identified by NBS were more likely to hospitalize children, especially when children exhibited signs or symptoms of the condition [59]. Over time parents of infants with false-positive CF results (identified as CF carriers) viewed their children as having very good or excellent health [79,81], though some reported feeling guilty for passing a mutation to their child [95].

Parents of infants with false-positive results repeatedly called for more factual information and guidance about the health implications for their child and family [35,63,65]. Tluczek et al. [95] found that some parents questioned the accuracy of false-positive results and believed their child might have CF, which led to increased vigilance for CF signs and symptoms. However, such concerns tended to abate over time when children remained healthy. While parents felt relieved that their child did not have CF, and empathy for parents of children diagnosed with CF, they also felt guilt for passing what they perceived to be defective genes to their offspring. Some took on new identities as “CF carriers”.

#### 3.3.4. Inconclusive or Intermediary Results

Follow-up diagnostic sweat tests for CF occasionally produce inconclusive or intermediary results. These include NBS results showing one or no CFTR mutations, with a sweat chloride level above the normal range but below a confirmatory CF diagnosis, or two mutations with a sweat chloride level within the normal range. Such results are classified as CF transmembrane conductance regulator-related metabolic syndrome (CRMS) in the US, and CF screen positive, inconclusive diagnosis (CFSPID) in Europe [110,111]. Most infants are healthy in early life, though some develop signs and symptoms of CF or receive a CF diagnosis later in childhood, and there are few data on long-term prognosis [110,111].

Parents of infants classified as having CRMS/CFSPID were uncertain about whether their child would develop CF-related health problems [55,93]. In one study, they reported lower levels of distress than parents of infants with a definitive CF diagnosis and considered their infants as healthy as parents of infants with negative NBS results [80]. By contrast, in another study, parents of children with this CRMS/CFSPID viewed their children as more vulnerable than parents of healthy children, but less so than parents of children with a CF diagnosis [100]. Parents struggled to understand uncertain results, worried about their infant’s health, and were vigilant for signs of CF [55,93]. Some reported frustration due to the lack of information about their child’s mutations and felt isolated because there were no national organizations or parent groups focused on intermediate results for CF [93].

#### 3.3.5. Comparisons across Groups

Studies that compared parent reactions to different types of NBS results showed similarities and nuanced differences based on the condition and results of confirmation testing. Parents of infants diagnosed with CF and those whose children had intermediate results were more worried than parents of infants who received normal sweat test results [29] or parents of healthy controls [80]. By contrast, another study found no differences in anxiety, depression, or stress between parents of infants with true-positive and false-positive results for CF [77]. Beucher et al. [27] compared the emotional responses of parents of infants identified as CF carriers to those of infants with persistent hypertrypsinemia but no CF mutations. Parents in both groups reported anxiety while awaiting confirmatory test results. However, at the two-year follow-up, some parents of children who were heterozygote carriers still expressed anxiety, particularly when their child was ill, while none of the parents of children with persistent hypertrypsinemia expressed concerns. Persistent anxiety was associated with lack of knowledge about CF, potential health implications for CF carriers, and transmission of CF mutations to future generations, as well as doubts about the accuracy of test results [27]. In another study, parents of infants with false-positive results for metabolic disorders who had other conditions reported the most stress, while parents in the false-positive/healthy group experienced anxiety while waiting for results, misunderstood the results, and expressed concern about the child’s health [73]. One study found that infants identified as CF carriers and infants with CF had more illnesses than the healthy comparison group [97]. However, it was unclear whether illness frequency in CF carriers was due to parents’ perceptions of child vulnerability or the physiological consequences of one CF mutation [97].

### 3.4. Parent Understanding of NBS Results

Research showed a link between parents’ understanding of test results and their levels of anxiety or stress. Gurian et al. [52] found that mothers who understood their child’s false-positive NBS results for metabolic disorders had lower levels of stress than mothers who did not understand the results. Many parents sought information prior to follow-up appointments from the internet, health care providers, family or friends, or books. Although source quality varied, those who accessed information prior to genetic counseling had higher levels of knowledge post-counseling [45,89,102].

Genetic counseling sessions improved understanding in many parents, though knowledge often declined over time [31,36]. In an intervention study, parents of infants with mutations for hemoglobinopathies who received genetic counseling were less anxious, had higher knowledge, and were more likely to discuss findings with other family members [63]. Carrier testing was also significantly higher among parents who received genetic counseling following false-positive NBS for CF [65,109]. Parents of infants with false-positive CF results who received information about the results also reported less anxiety and depression [106]. Receiving tailored written and video-recorded information two weeks after genetic counseling significantly improved parents’ understanding of their child’s CF carrier status [81].

However, even with genetic counseling, some parents of children identified as carriers for CF or SCD mistakenly believed their infant might develop the condition [47], or were confused about the implications of the test results [67]. Parents of infants with false-positive results for CF had difficulty understanding the genetics of autosomal recessive conditions [36]. In another study, about three-quarters of mothers of infants at increased risk for T1D accurately reported their infant’s risk shortly after receiving NBS results, but over time this rate declined to two-thirds [31]. Difficulty retaining and understanding information from genetic counseling sessions was attributed to parental anxiety and concerns about the child’s health [67].

### 3.5. Parent Education following Confirmed Diagnosis

Parent education is critical following a confirmed diagnosis. Jessup et al. [57] found that parents wanted to know the severity of the condition, effect on their child’s life, and practical information about keeping their child well, followed by education throughout the child’s first year of life. Parents also welcomed messages that engendered confidence in their ability to care for their child and conveyed hopefulness for their child’s future. They advocated for adapting educational approaches to each family, considering parents’ life experiences, knowledge, education, and family circumstances. Timing and mode of parent education affected parents’ desire to engage with care teams and their ability to retain information. Parents may need a few days to absorb the diagnosis before participating in intensive education. Sawyer and Glazner [84] compared parents’ experiences of residential education and outpatient teaching following a CF diagnosis. While residential programs saved repeated travel to and from the facility, they also had to take time off from work and find childcare for siblings. Despite the challenges, all parents said they would recommend the residential program.

### 3.6. Communication

#### 3.6.1. Provider-Parent Communication

Parents’ preferences for communication with providers varied depending on the condition and type of results and may reflect perceptions about the seriousness of the condition. For example, parents of children with positive results for CF preferred in-person disclosure, while parents of infants with CH were comfortable with disclosure by phone [83]. Parents of infants diagnosed with CF or SCD who received false reassurance of likely negative NBS results from their primary care providers experienced distress when results were positive [34]. Being notified by letter of positive NBS results for CF or SCD was also distressing, as letters failed to provide adequate explanations about test results or reasons for required follow-up [58]. If the provider was known, telephone communication was acceptable, but face-to-face communication was preferred for unfamiliar providers. Follow-up phone calls were helpful for parents of infants with CF or SCD and those who received positive NBS results, providing opportunities to answer questions, clarify misunderstandings, and learn about other resources [64,91].

Many factors contributed to the quality of provider-parent communication. For parents, limited knowledge of NBS, misunderstanding screening tests, emotional distress, and having only one parent attend the appointment impeded communication [44]. Parents preferred to receive initial results from professionals knowledgeable about NBS and the condition for which their infant screened positive and who could communicate clearly and empathically. Ideally, such individuals would be known, trusted providers, though being knowledgeable superseded familiarity [22,45,51,73,87,88,93,94]. Parents wanted providers to communicate factual information in simple, jargon-free language with sensitivity to parents’ emotional state [22,35,36,91,94]. Receiving NBS results by voicemail, or before weekends or holidays, was extremely troubling because questions and concerns could not be addressed in a timely manner [49,59,94]. Disruptions during genetic counseling sessions also adversely affected parents’ recall [43].

#### 3.6.2. Informing Children of Their Carrier Status

Parents of infants identified as carriers for a genetic condition faced the additional dilemma of when and how to inform their children about the NBS results. Parents believed that their children had a right to know their carrier status, but worried that this information might damage the child’s self-esteem and adversely impact their child’s opportunities to find a partner and have a family [32,103]. Consequently, parents wanted to emphasize that the child was “normal” and that no one is to blame [103].

Parents also carefully considered the timing and approach to informing their child about their carrier status and its potential impact on the child’s reproductive future. They wanted additional support and guidance as they navigated the communication process [95,104].

A subset worried about stigma or rejection from potential partners [95]. Some tried to normalize their child’s carrier status by making the telling part of the child’s birth story. Others identified strategic opportunities, such as when children learned about genetics, when teens began dating, or when young adult children began planning their own families [32,103]. Some parents planned to inform their children themselves, believing that they know their children best, while others welcomed the assistance of knowledgeable health professionals [103].

#### 3.6.3. Communicating NBS Results to Family Members

Parents of children with CF were comfortable sharing the diagnosis with family members and friends, but reported difficulty answering questions about genetics. In the same study, parents of children with SCD were more cautious about sharing the diagnosis for fear of stigma and discrimination [34]. New genetic information affected extended family relationships, as some parents wondered if family members might have CF or be carriers. This posed moral dilemmas for some parents, who needed to decide whether and how to share genetic information with biological relatives who might be at risk for having a child with the condition. Sharing genetic information with extended family was an opportunity for some and a burden for others, depending on relationship quality. Parents were concerned that the information could provoke anxiety in at risk family members. Such decisions were particularly challenging when parents had strained relationships with extended family members [95,104]. Many parents chose not to share their child’s CF carrier status with health care providers, health insurance companies, children’s classmates or teachers or their spiritual leaders [67].

### 3.7. Parent Decisions about Carrier Testing and Future Pregnancies

The impact of genetic NBS results on carrier testing uptake and future reproductive decisions varied based on type of result and conditions identified [78]. Following positive screens for CF and SCD, parents expressed interest in and uptake of carrier testing; however, higher anxiety among mothers of infants identified as SCD carriers was associated with genetic test avoidance [22]. Concerns about recurrence prompted many parents to decide against having more children. Others obtained prenatal testing for subsequent pregnancies, and some opted to terminate affected pregnancies [46,73,78,80,85,87]. Interestingly, one study showed that parents of infants with false-positive results for metabolic or endocrine disorders were more likely to consider not having more children than parents of infants with true-positive results [73]. In another study, positive NBS results for CF prompted some parents to have subsequent prenatal testing, in order to choose to terminate an affected pregnancy or prepare to have a child with special needs [85]. In the same study, reasons for not pursuing prenatal testing included disapproval of termination, belief that the condition was not serious, or concerns about health risks associated with prenatal testing.

### 3.8. Child and Family Outcomes

#### 3.8.1. Parent-Child Relationships

The impact of NBS results on parents’ relationships with their children and perceptions of their vulnerability varied. Parents of infants diagnosed with CF were “excessively protective and indulgent” of affected children, while relationships with infants who had inconclusive results were less affected [80]. Parents of infants with intermediate CF results reported “delayed infant bonding” and changes in parenting behavior, though these issues abated over time [55]. However, other studies found no differences in self-reported rejection or protective parenting behaviors among mothers of infants diagnosed with DMD or CF or those identified as CF carriers, compared to mothers in the general population [79]. Parents of infants diagnosed with DMD or CF did not believe that the diagnosis would influence their relationship with or their plans for raising their child [35,40].

Anxiety and stress due to confusion about test results adversely affected parents’ interactions with their children [53,63,66]. In two studies [52,101], parents of infants with false-positive results for metabolic disorders perceived their children as being vulnerable to illness, which was associated with parental overprotectiveness and a focus on physical symptoms. Compared to parents of infants with normal NBS, these parents reported more challenging interactions with their children and higher levels of parenting stress, and described their children as being more difficult and requiring more care [52]. Mothers of children diagnosed with CF were more likely to be protective of their children, compared to mothers of infants with positive NBS for other conditions [34,36,72]. Parents who perceived their child as vulnerable were also more likely to view their child as being less attached than parents who identified their children as less vulnerable [99]. Parents of pre-school children at increased risk of T1D perceived their children as vulnerable to illness and altered their parenting behavior accordingly [60]. However, by age 12 there were no differences between low and high risk groups in parenting styles [62]. Parents of 12-year-olds were also more likely to underestimate their child’s risk, compared to when their children were infants, which could affect parenting behavior. By contrast, Baughcum et al. [25] found that family history of T1D and high maternal anxiety led parents to engage in more prevention behaviors. While some were beneficial (i.e., watching for signs of diabetes, providing healthy foods) [25,61], others were potentially harmful (i.e., limiting contact with other children, administering insulin unnecessarily) [25]. Some parents also chose not to share risk-related information with children who were at increased risk for T1D [61]. In another study [94], mothers of infants diagnosed with CF through NBS were less likely to breastfeed than mothers of infants with normal NBS. Bottle feeding was also associated with less responsive and more task-oriented feeding behaviors than breastfeeding. This has implications for maternal bonding and child health because of the significant immunologic properties of human milk [112]. Another study found that parents of infants with CH and those in a healthy control group had similar interactions with their infants, compared to parents of infants diagnosed with CF or identified as CF carriers, suggesting the severity of the condition identified through NBS may influence parents’ perceptions of and relationships and interactions with their infants [97].

#### 3.8.2. Child Development, Family Burden, and Quality of Life

Effects of positive NBS results on child development, family burden, and quality of life varied depending on the disorder identified, the child’s age, and the outcomes measured. School-aged children with CH diagnosed though NBS were at increased risk for behavioral problems, which dissipated as children became more psychologically mature [28]. Similarly, although young adults with CH diagnosed through NBS showed lower health-related quality of life, they had no significant differences in happiness, autonomy, psychosexual development, gross motor function, educational attainment, or marital status compared to healthy peers [105]. Parents of children with metabolic disorders identified through NBS [50] reported that the child’s developmental delays contributed to family burden and parental stress. Despite these challenges, parents maintained positive expectations about their children’s developmental capacities and futures. Parents of children diagnosed with metabolic disorders through NBS also reported improved child development and less parenting stress than parents of children diagnosed clinically [107,108], while parents of children with DMD diagnosed through NBS reported similar family quality of life to parents of children diagnosed clinically [35]. Studies found no differences in health-related quality of life or psychosocial function in youth with CF diagnosed through NBS compared with those diagnosed clinically [96,98]. However, parents of youth with CF diagnosed through NBS were at increased risk of depressive symptoms [98]. Parents of infants at increased risk of T1D reported no significant concerns about their children’s psychosocial development or self-concept twelve years after neonatal identification of T1D risk [62].

### 3.9. Disparities

Several studies uncovered disparities based on parents’ age, race, marital status, insurance, immigration status, and primary language. Postpartum women who were predominately African American had significant gaps in their general knowledge of NBS [66]. Another study found that parents who were single, non-white, had less than a college education, did not have private health insurance, and had their children early in life lacked knowledge about false-positive NBS results for CF [67]. Black mothers were less likely to allow their newborns to be screened for FXS than Hispanic or white mothers, possibly reflecting cultural differences or historical mistrust of health care systems [88].

Sociodemographic factors also influenced parents’ outcomes. Maternal risk for anxiety [26] or depression [56] following identification of increased risk of T1D was associated with being single, of Hispanic ethnicity, low education, and/or having female infants. Although most parents were concerned about their infant’s positive NBS results for CF, parents of first-born children and those who were immigrants were even more worried [29]. In another study, African American and Hispanic mothers and those with less than a college education were least likely to accurately report their infant’s risk for developing T1D [31]. Spanish-speaking and bilingual parents living in the US also identified language barriers to learning about NBS [39]. Providers seldom spoke Spanish and most written information was only available in English. In Spanish, NBS is referred to as “blood test” and can be easily confused with other blood-based tests performed on newborns. Parents who had low health literacy and identified as multiracial reported more dissatisfaction with their NBS experience [47]. Younger parents of infants found to be SCD carriers, and parents of CF carriers who were biracial or multiracial, were more likely to have misconceptions about NBS results.

Parental knowledge and acceptance of NBS results also varied according to sociodemographic characteristics. Parents who had never heard of CF were more likely to have less education, speak English as a second language, and were less likely to have private insurance [48]. In one of the few studies comparing fathers and mothers, fathers were more likely to be completely uninformed about NBS [92], which likely amplified their emotional reactions to receiving positive NBS results. Mothers with lower incomes were almost four times less likely to receive information prenatally and more likely to be informed during the suboptimal postpartum time than their higher income counterparts [92]. Additionally, parents of varied demographic backgrounds expressed concerns about potential societal stigma and discrimination associated with the diagnostic labels of “cystic fibrosis” and “sickle cell disease” or being “carriers” of genetic mutations for either of these conditions [22,56,62,71,111].

## 4. Discussion

This comprehensive systematic review synthesizes twenty years of research that investigated psychosocial issues associated with NBS at the dawn of genomic technologies. Despite the global increase in NBS programs over the last five decades, many new parents remain uninformed about the purpose of NBS and the potential implications of positive results for their families. Findings support expanded, comprehensive parent education that includes information about use of DBS for research.

### 4.1. Parents’ Psychosocial Response to NBS

Although the optimal time and approach to educating parents about NBS is still under debate, parents clearly believed receiving comprehensive information was important. Those who had already experienced NBS advocated for offering information before and during pregnancy, as well as at the time of specimen collection. Communicating information in multiple ways is also helpful. Results of a randomized controlled intervention found that women who received information via videos and brochures during the last trimester of pregnancy had significantly greater knowledge of and more positive attitudes about NBS and storage of DBS for research than the control group, which received only brochures [113]. Clinicians need to carefully time postpartum education to be sure that parents are sufficiently rested and alert to comprehend and retain information. In every interaction, clinicians must take time to discuss the purpose of NBS, conditions identified, when and how to expect results, the meaning of positive results, and the relatively low risk of receiving positive results [92]. Improving parents’ awareness and understanding of NBS may also reduce the shock they feel when results are positive.

Regardless of the condition, a positive NBS result is associated with a range of parental emotions, including relief, guilt, shock, and denial [34,83]. Kerruish et al. [16] suggest that research might underestimate the extent of parental distress, because the most distressed parents might have declined to participate. Parents’ emotions may not be reflected in standard measures of anxiety and stress, highlighting the value of qualitative approaches that elicit their thoughts and feelings associated with their child’s NBS results. Findings underscore a link between parental distress and misunderstanding of NBS results, particularly false-positive results; however, education and counseling can lessen persistent confusion and anxiety. Early involvement of specialists, reducing wait time for confirmatory testing, and continued monitoring by trusted providers can reduce parental anxiety following positive NBS.

Research suggests that neonatal diagnosis of serious health conditions could impact parents’ perceptions of their child’s vulnerability and alter their approach to parenting. Emphasizing the benefits of normative childhood experiences, peer relationships, and pastimes is an important element of anticipatory guidance for families following diagnosis of potentially serious conditions or false-positive results through NBS. Comprehensive multidisciplinary care and enhanced psychological and social support can also contribute to positive family outcomes.

### 4.2. Communication of NBS Results

We encourage clinicians to assess parents’ emotional states and most immediate concerns as part of every communication of positive NBS results. In our intervention work [114], asking parents to rate their level of worry on a scale of 1–10 at the beginning of a genetic counseling session for follow-up sweat testing for a positive CF screening result was an efficient way to gauge their distress. We then inquired about what parents were “most worried about” and immediately addressed that issue. Reducing some of parents’ initial distress may improve their capacities to receive new information.

We agree with the conclusions of Buchbinder and Timmermans [30] that effectively communicating “bad news” to new parents continues to challenge clinicians. Despite providers’ diligent efforts to explain NBS results to parents, parents remained confused about test results and related implications. A clear deficit in parental knowledge of the rationale for follow-up testing of positive results was evident in this review. Thus, we urge clinicians to explain the difference between “screening” tests and “diagnostic” tests so parents will understand the rationale for additional testing following positive NBS results.

Table 4 lists recommendations based on available research to improve parents’ comprehension and retention of test results and their experiences with NBS. Parents’ retention of information received during counseling sessions could be improved by providing additional resources before, during, or after sessions. These could include packets of written information or access to educational videos [81,89]. Parents who found information on their own, before counseling, were more likely to retain information received during the session [89]. Unfortunately, such information was generally not offered to parents before follow-up appointments, and its quality cannot be assured, as information was found by parents, not professionals. Systematically sending information to parents before genetic counseling sessions might improve their short and long-term understanding of critical NBS facts. Given the multi-step process required for establishing or ruling out a diagnosis following a positive NBS, effective communication and ongoing education throughout the process are critical to successful NBS programs. Collaboration among regional laboratories, specialists, and primary care providers and effective provider–parent communication are paramount for ensuring parents understand NBS results and for mitigating their distress following positive results.

### 4.3. Molecular Genetic Technologies in NBS

Evidence highlights the unique nature of NBS results that rely on molecular genetic technologies that can identify infants who are carriers of recessive conditions such as CF and SCD, or at risk for developing conditions like T1D, as well as those with health conditions. These results also provide information about parents’ carrier status for recessive conditions. While this unsolicited information can be critical for future reproductive decisions, it also creates challenges in sharing the implications with their children and extended family members. Use of molecular genetics in NBS was largely responsible for identifying the broad spectrum of CF phenotypes now classified as CRMS/CFSPID. While these new discoveries offer the benefits of improved clinical monitoring, they also impose a psychosocial burden of prognostic uncertainty.

Parents typically rely on clinicians to help them understand and apply these complex findings in their own families. Communicating genetic test results requires translation of complicated genetic concepts and understanding of the sociocultural context in which parents receive such information, making genetic counseling absolutely imperative.

Knowledge about the implications of heterozygosity for recessive conditions is expanding rapidly. Heterozygote carriers of some recessive mutations may show some clinical signs and symptoms of the condition. For example, those with one copy of a mutated allele for SCD, an autosomal recessive condition, may demonstrate the effects of the mutation under certain environmental conditions [115]. In another example, children who are heterozygote carriers of a CF mutation may receive NBS results indicating that they are unaffected carriers, or that they have inconclusive NBS results. Children in either group could develop symptoms in the future [115].

Although expanded genetic NBS that identifies potential risk for developing later-onset disorders is currently limited, whole-genome or whole-exome sequencing for NBS is likely to become more common. The implications of this expansion are immense and raise important questions for parents, clinicians, researchers, and policymakers. Exploring stakeholders’ perspectives, particularly parents’, on the use of genomic sequencing for NBS is essential to ensure ethical and equitable future policies [116,117]. Our review suggested that genetic NBS to determine T1D risk may have less public support than conditions that manifest early in life [61]. By contrast, another recent report noted that parents and the public may view genomic data as empowering and important. Yet, providers may be more reticent to employ broader genomic testing that provides substantial information about future disease risk and may find it challenging to explain such information to parents [118]. Concerns about the utility of genomic sequencing for underrepresented populations and issues of privacy remain [118,119]. Also of paramount importance is equitable access to genetic counseling resources [116].

Clinicians must ensure that parents receive up to date information about NBS and that they understand the potential implications for their child, themselves, and other family members. This information must be presented in ways that do not provoke undue psychological distress. As each family is unique, the amount and type of information and the sequence in which it is presented should be tailored to match parents’ needs. For example, evidence shows that during follow-up testing for CF, parents’ preferences may differ depending on their perception of the child’s risk of actually having CF. Parents may also not be ready to receive all relevant information immediately after receiving test results. Clinicians must remain cognizant that potentially upsetting NBS results were not solicited by parents and are often shared at a time when they may be sleep deprived and overwhelmed by caring for their newborn. Some may also suffer from postpartum depressive symptoms, which can be exacerbated by receiving positive NBS results. These factors can impair parents’ capacities to absorb and assimilate new information.

This review also identified disparities in understanding among marginalized subpopulations, which further supports the need for clinicians to tailor information to parents’ existing knowledge and immediate needs, involve professional interpreters in counseling, reinforce information offered at the initial counseling session, and provide ongoing access to additional counseling. Parents of newly diagnosed infants with CF valued initial genetic counseling sessions, but did not identify genetic information as the most important information received at the time of diagnosis [84]. Thus, clinicians also need to adjust the content of each educational or counseling session to accommodate parent preferences. Results of genetic NBS also have important implications for other children in the family. NBS is a process with a series of integrated procedures. For this reason, it is imperative that genetic counselors support long-term follow-up of genetic NBS results [116].

### 4.4. Limitations

Most studies were conducted in high resource countries in North America and Europe. Most used cross-sectional designs with non-standardized researcher-designed assessments without documented psychometric properties, relied on convenience samples that were often small and homogenous (i.e., mostly white educated mothers when demographic data were documented), and lacked a theoretical basis. There were only six intervention studies. Few studies included participants who spoke a language other than the national language of the country in which the study was conducted. Despite a proliferation of NBS programs that identify heterozygote carriers of autosomal recessive conditions, only two studies examined parents’ perspectives about or experiences of communicating this information to their children. Parents’ reasons for supporting NBS were articulated in multiple studies, but opposition to or ambivalence about NBS were seldom explored, suggesting researcher bias in favor of NBS. The majority of studies also focused on NBS for CF and SCD, with few comparisons across conditions. Molecular genetic screening tests for these conditions, which detect carriers, were the first such tests introduced in practice, which is likely why most research has focused on them. Studies should be expanded to include a greater diversity of conditions, to determine if results are consistent across conditions. Authors acknowledge that there could have been research articles not captured by the search and screen process used for this report.

### 4.5. Implications for Future Research

Future investigations must be more inclusive of culturally and socioeconomically diverse families and should examine psychosocial effects of NBS in low-resource countries. Researchers need to design interventions that explicitly identify ways to address the informational needs of families with low health literacy and those in marginalized populations. We agree with O’Connor et al. [77] that further research should focus on the “marital relationship, paternal NBS experiences and continual parental education”. Research must also establish evidence-based guidelines for timing and modes of communication about NBS and DBS retention to optimize parents’ comprehension and retention of information. We agree with Farrell et al. [115] that, while there may be health and psychosocial benefits to identifying carriers of recessive conditions, it is imperative to explore parents’ reasons for receiving or not receiving such information and at what time this information should be shared, if at all. Such research is essential to informing counseling approaches that respect parents’ preferences while ensuring their comprehensive understanding of the implications for their child, themselves, and for future pregnancies. Process research using video-recorded counseling sessions can identify communication skills that effectively address parents’ needs for emotional support while providing factual information. Such research can inform assessment techniques that guide clinical judgment regarding when and how much information and/or emotional support to offer parents. Finally, empirical evidence is needed to help guide parents in deciding when and how to inform and educate their children about their genetic status and related reproductive implications.

## 5. Conclusions

The psychosocial consequences of an unexpected neonatal diagnosis and unsolicited genetic information remain significant risks to NBS. Adverse sequalae may be reduced by providing comprehensive, multimodal parent education about NBS throughout pregnancy and the postpartum period, delivering tailored genetic counseling to parents, and offering anticipatory developmental guidance and support for families receiving positive NBS results. Clinicians must recognize the unique needs of families with marginalized identities and take measures to ensure equitable service delivery. Future intervention research needs to be more inclusive of culturally and socioeconomically diverse families as well as families from low-resource countries. Finally, it is critical that resources be allocated to low-income nations and disenfranchised communities to achieve equitable access to NBS.

## Figures and Tables

**Table 1:** Databases and Search Terms.

Database	PubMed	PsycINFO	CINAHL
Search Terms	(“Neonatal Screening” [Mesh] OR “newborn screening” OR “neonatal screening”) AND (“Reproductive Behavior” [Mesh]) OR “Reproductive decision” OR “Communication” [Mesh] OR (“Knowledge” [Mesh] OR “Knowledge of Results, Psychological” [Mesh]) OR (“Attitude” [Mesh] OR “Attitude to Health” [Mesh]) OR “Quality of Life” [Mesh] OR “Adaptation, Psychological” [Mesh] OR “Emotional Adjustment” [Mesh] OR “Social Stigma” [Mesh] OR “Scapegoating” [Mesh] OR “Family Relations” [Mesh] OR “Stress, Psychological” [Mesh] OR “Psychological Distress” [Mesh] OR “Parents/psychology” [Mesh] OR “test results” OR “Psychosocial Support Systems” [Mesh] OR stress [tiab] OR distress [tiab] OR stigma [tiab] OR stigmatiz* [tiab] OR blame OR attitude OR knowledge [tiab] OR psychosocial [tiab] OR emotion * OR communication [tiab] OR “quality of life” OR “decision making” [tiab] OR “decision-making” [tiab] AND (mother [tiab] OR father [tiab] OR parental [tiab] OR parent [tiab])	DE “Parent Child Relations” OR DE “Parental Attitudes” OR DE “Parental Involvement” OR DE “Parental Attitudes” OR (DE “Mother Child Relations” OR DE “Mothers” OR (DE “Father Child Relations” OR DE “Fathers”) OR parent* OR mother OR father OR spousal AND stress OR distress OR stigma OR stigmatiz* OR blame OR attitude OR knowledge OR psychosocial OR emotion* OR communication OR “quality of life” OR “decision making” OR “decision-making” OR psychological OR “reproductive decision” AND TI (screen OR test) AND (newborn OR neonate) OR AB (screen OR test) AND (newborn OR neonate)	(neonate OR newborn OR neonatal OR infant) AND (“blood test” OR “neonatal screening” OR “newborn screening”) AND (stress OR distress OR stigmatiz* OR “psychological harm” OR blame OR dynamics) AND (parent OR parental OR family OR families)
Filters	English, from 2000–2020	English, from 2000–2020	English, from 2000–2020
Results	296 articles	220 articles	55 articles Total 571

**Table 2:** Inclusion/Exclusion Criteria.

Study focus	Include: psychosocial issues related to NBS Exclude: prenatal screening, studies about diagnostic tests (not NBS), genetic testing unrelated to NBS, biomedical research (e.g., sensitivity and specificity of tests)
2. Participants	Include: parents and their infants who underwent NBS Exclude: extended family
3. Children’s conditions	Inclusion: any condition identified through NBS blood samples Exclusion: conditions not identified through NBS or do not use blood samples (e.g., congenital heart conditions, hearing problems)
4. Timeframe for data collection	Include: any time post NBS Exclude: prenatal data collection
5. Participant age	Inclusion: any child age, any parent age related to NBS Exclusion: unrelated to NBS
6. Countries	Include: all Exclude: none
7. Language	Include: report written in English Exclude: report only available in language other than English
8. Pub Year	2000 through 2020
9. Study Design	Include: qualitative, quantitative, and mixed methods studies that used parent self-report and/or observations by others Exclude: systematic or narrative reviews, opinion papers, study proposals
10. Sources	Include: primary sources in peer-reviewed journals Exclude: secondary sources, articles in non-peer reviewed sources, dissertations

**Table 3:** Study Characteristics.

First Author (Year) Location	Purpose	Design Data Collection Method	Condition(s) Screened	Parent Sample Size % (*n*)	Child Sample Size
Ahmad et al. (2014) USA [22]	Assess emotion-related language in mothers’ narratives about NBS and parenting behavior	Cross-sectional mixed methods Interviews Researcher designed questionnaire Standardized assessments	SCD carriers	187 mothers	187 infants
Araia et al. (2012) Canada [23]	Identify associations between pre-NBS education and parents’ knowledge of and satisfaction with NBS	Cross-sectional quantitative Researcher designed questionnaire	Negative NBS results	750 mothers	750 infants
Bailey et al. (2015) USA [24]	Assess mothers’ reactions to disclosure of their infants’ FXS carrier status identified through NBS	Longitudinal quantitative Standardized assessments	FXS carriers	33 mothers 54.5% (18) mothers of FXS carriers 45.4% (15) mothers of negative NBS	18 FXS carriers 15 negative NBS
Baughcum (2005) USA [25]	Assess mothers’ preventive efforts in children identified through NBS as being at risk for T1D	Longitudinal quantitative Semi-structured interviews	Increased risk of T1D	192 mothers	192 infants 7% (13) very high risk 37% (71) high risk 56% (108) moderate risk
Bennett Johnson et al. (2004) USA [26]	Describe maternal anxiety associated with NBS for T1D	Longitudinal mixed methods Interviews Standardized assessments	Increased risk of T1D	435 mothers	435 infants 5.3% (23) very high risk 34.7% (151) high risk 60% (261) moderate risk
Beucher et al. (2010) France [27]	Evaluate long-term effects on parents of infants with false-positive NBS for CF who were heterozygote CF carriers (HZ) compared to infants with persistent hypertrypsinemia (PHT)	Longitudinal mixed methods Interviews Standardized assessments	False-positive CF results (HZ, PHT)	86 parents 72.1% (62) HZ (1 father) 27.9% (24) PHT	86 infants 72.1% (62) HZ 27.9% (24) PHT
Bisacchi et al. (2011) Italy [28]	Assess psychological adjustment of children with CH compared to peers without CH	Cross-sectional quantitative Standardized assessments	CH	168 families 84 CH 43.4% (73) mothers 36.3% (61) fathers 84 healthy 50% (84) parents healthy	84 CH 84 healthy
Brockow and Nennstiel (2019) Germany [29]	Evaluate parents’ experiences of positive NBS for CF	Cross-sectional quantitative Researcher designed questionnaire	CF	105 parents 62.7% (64) mothers 7.8% (8) fathers 29.4% (30) both parents 2.9% (3) missing data	105 infants 68.8% (72) CF negative 25.7% (27) CF 5.7% (6) CFSPID
Buchbinder and Timmermans (2012) USA [30]	Describe parents’ experience of clinical communications about positive NBS results for metabolic disorders	Cross-sectional qualitative Semi-structured interviews Observations	Metabolic disorders	75 families observed 27 interviewed 74.1% (20) mothers 3.7% (1) grandmother 11.1% (3) fathers 11.1% (3) couples	75 infants 12% (9) false-positive 32% (24) true-positive 56% (42) ambiguous results
Carmichael et al. (2003) USA [31]	Assess mothers’ understanding of their newborns’ genetic risk for T1D	Longitudinal mixed methods Structured Interview Standardized assessments	Increased risk of T1D	435 mothers	435 infants 60% (261) moderate risk 34.7% (151) high risk 5.3% (23) very high risk
Cavanagh et al. (2010) USA [32]	Assess long-term impact of genetic counseling following false-positive NBS for CF	Cross-sectional mixed methods Structured Interview	False-positive CF NBS	37 parents	37 children
Christie et al. (2013) Australia [33]	Evaluate the feasibility and acceptability of NBS for fragile X syndrome (FXS)	Cross-sectional mixed methods Researcher designed questionnaire	Fragile X syndrome	1971 mothers	2000 newborns
Chudleigh et al. (2016) England [34]	Explore parents’ experiences of receiving positive NBS result for CF or SCD	Cross-sectional qualitative Grounded theory Interviews	CF SCD	12 families 100% (12) mothers 83.3% (10) fathers	12 infants 41.6% (5) CF 58.3% (7) SCD
Chung et al. (2016) USA [35]	Compare parents’ attitudes about NBS for Duchenne muscular dystrophy (DMD) and Becker muscular dystrophy (BMD) between diagnosis by NBS vs. clinical symptoms	Cross-sectional quantitative Researcher designed questionnaire	DMD BMD	25 parents 68% (17) mothers 32% (8) fathers	15 males
Ciske et al. (2001) USA [36]	Evaluate effectiveness of risk communication regarding infant CF carrier status identified through NBS	Cross-sectional mixed methods Researcher designed questionnaire Semi-structured interviews	CF carriers	138 parents questionnaires 123 parents interviewed	138 children
Collins et al. (2013) USA [37]	To study how parent’s experience and reactions are influenced by various factors during initial NBS carrier results disclosure	Cross-sectional mixed methods Semi-structured interviews	CF carriers SCD carriers	270 parents 36.6% (99) parents of CF carriers 63.3% (171) parents of SCD carriers	
Davey et al. (2005) Australia [38]	Examine mothers’ knowledge of NBS and attitudes about retaining blood samples for research	Cross-sectional quantitative Researcher designed questionnaire	NBS in general	600 mothers	
Davis et al. (2006) USA [39]	Evaluate parent and provider knowledge of NBS, opinions from parents about content and timing of NBS education to develop recommendations	Cross-sectional qualitative Focus groups (22) Individual interviews (3)	Positive or false-positive NBS results	138 individuals 37% (51) parents 56.5% (78) health care providers 6.5% (9) state NBS professionals	
de Monestrol et al. (2011) Sweden [40]	Assess parents’ attitudes about NBS for CF and potentially learning their own carrier status	Cross-sectional quantitative Researcher designed questionnaire	CF	719 parents 16.8% (121) parents CF 41.6% (299) parents diabetes 41.6% (299) parents healthy	719 children and adults 16.8% (121) CF 41.6% (299) diabetes 41.6% (299) healthy
DeLuca et al. (2011) USA [41]	Explore parents’ experiences of diagnostic evaluations for metabolic disorders recently added to NBS	Longitudinal qualitative descriptive Semi-structured interviews	Metabolic disorders	30 families 48 interviews 12 mothers 18 couples (both parents)	30 infants with positive NBS for metabolic disorders
Dillard and Carson (2005) USA [42]	Improve understanding of how family members and HCPs manage uncertainty following NBS for CF	Cross-sectional qualitative Videotaped clinic interactions	CF	17 families	17 infants with positive NBS for CF
Dillard et al. (2007) USA [43]	Assess the effects of disruptions during genetic counseling on parent recall of genetic risk information	Cross-sectional quantitative Researcher designed questionnaire Videotaped clinic interactions	False-positive CF NBS (all carriers)	20 families 53% (21) mothers 45% (18) fathers 3% (1) grandmother	20 infants with false-positive NBS for CF; all CF carriers
Dillard et al. (2008) USA [44]	Examined potential threats to effective risk communication with parents of infants with received positive NBS for CF	Cross-sectional mixed methods Researcher designed questionnaire Videotaped clinic interactions	CF	17 families 100% (17) mothers 64.7% (11) fathers 29.4% (5) grandmothers	17 infants with positive NBS for CF
Dillard et al. (2010) USA [45]	Examined parent information-seeking behavior prior to and during clinic visit related to their infant’s positive NBS for CF	Cross-sectional exploratory mixed methods Researcher designed questionnaire Videotaped clinic interactions	CF	20 families 100% (20) mothers 85% (17) fathers 3% (1) grandmother	20 infants with positive NBS for CF
Dudding et al. (2000) Australia [46]	Document women’s reproductive decisions after NBS identified CF	Cross-sectional quantitative Researcher designed questionnaire	CF	124 mothers	124 children with CF
Farrell et al. (2020) USA [47]	Assess parents’ experiences of communication about NBS that identified infant as carrier of SCD or CF	Cross-sectional mixed methods Researcher designed questionnaire	CF carriers SCD carriers	714 parents 59.7% (426) parents SCD carriers (98.1% [418] mothers) 40.3% (288) parents CF carriers (96.5% [278] mothers)	714 infants 59.7% (426) false-positive NBS for SCH 40.3% (288) false positive for CF
Fitzgerald et al. (2016) Ireland [48]	To evaluate maternal understanding of NBS for CF, and awareness of clinical features of CF	Cross-sectional quantitative Researcher designed questionnaire	NBS in general CF	1142 women 58% (662) antenatal 42% (480) postnatal	
Fitzpatrick et al. (2019) Ireland [49]	Examine parent awareness of NBS and conditions screened	Cross-sectional quantitative Researcher designed questionnaire	CF	124 parents CF 662 antenatal women 480 postnatal women	
Gramer et al. (2014) Germany [50]	Examine parents’ perspectives about their child’s development, future expectations, and family burden related to metabolic disorders identified through NBS	Cross-sectional quantitative Researcher designed questionnaire Developmental assessment	Metabolic disorders	187 parents	187 children
Grob et al. (2008) USA [51]	Explore parents’ experiences of NBS and impact on interactions with other family members and providers	Cross-sectional qualitative Semi-structured interviews	CF	35 parents 94.2% (33) mothers 5.7% (2) fathers	35 infants
Gurian et al. (2006) USA [52]	Assess impact of false-positive NBS results on parents of children tested for an expanded panel of metabolic disorders	Cross-sectional quantitative Researcher designed questionnaire Standardized assessments	False-positive NBS on expanded panel for metabolic disorders	356 parents 65.4% (233) mothers 71.7% (167) false-positive 28.3% (66) normal NBS 123 fathers 70% (86) false-positive 30% (37) normal NBS	240 infants 72.1% (173) false-positive 27.9% (67) normal NBS
Hayeems et al. (2016) Canada [53]	Assess the psychological impact of false-positive NBS result for CF on parents	Cross-sectional mixed methods Researcher designed questionnaire Standardized assessments Semi-structured interview	False-positive results for CF	544 mothers 24.6% (134) false-positive 75.4% (410) normal NBS	544 infants 134 false-positive 410 normal NBS
Hayeems, Miller et al. (2017) Canada [54]	Assess health care use among families with false-positive NBS results for CF	Cross-sectional quantitative Database Analysis	False-positive results for CF	7820 mothers 20% (1564) false-positive 80% (6256) negative NBS	7820 infants 20% (1564) false-positive 80% (6256) negative NBS
Hayeems, Miller, Barg et al. (2017) Canada [55]	Examine psychosocial consequences of diagnostic uncertainty resulting from NBS for CF	Cross-sectional mixed methods Researcher designed questionnaire Standardized assessments	CF+ Inconclusive for CF	Parents of 442 infants Quantitative 442 mothers 3.4% (15) CF 3.8% (17) inconclusive 92.8% (410) normal NBS Qualitative 20 parents 100% (20) mothers 10% (2) fathers	442 infants 3.4% (15) CF 3.8% (17) inconclusive 92.8% (410) normal NBS
Hood et al. (2005) USA [56]	Assess maternal depression related to infant’s risk for T1D identified through NBS	Cross-sectional quantitative Standardized assessments	Increased risk for T1D	192 mothers 100% (192) 1st interview 75% (144) 2nd interview	192 infants 29% high risk 71% moderate risk 0.6% very high risk
Jessup et al. (2016) Australia [57]	Understand parents’ experience of their initial education following their infant’s CF diagnosis resulting from NBS	Cross-sectional qualitative Interviews	CF	10 parents of 7 children 100% (7) mothers 42.9% (3) fathers	7 children
Johnson et al. (2004) USA [8]	Describe maternal anxiety during the 1st year following notification of infant’s genetic risk for T1D	Longitudinal mixed methods Structured interviews	Increased risk for T1DM	435 mothers 60% (261) moderate risk 34.7% (151) high risk 5.3% (23) very high risk	435 infants 60% (261) moderate risk 34.7% (151) high risk 5.3% (23) very high risk
Kai et al. (2009) England [58]	Understand parents’ experiences of being informed of NBS results that identify their infant as a CF or SC carrier	Cross-sectional qualitative Semi-structured interviews	CF carriers SCD carriers	67 family members 73.1% (49) mothers 23.9% (16) fathers 1.5% (1) maternal grandmother 1.5% (1) maternal grandfather	51 infants 53% (27) CF carriers 47% (24) SCD carriers
Karaceper et al. (2016) Canada [59]	Evaluate impact of false-positive NBS results for metabolic disorders on health care utilization	Cross-sectional quantitative Database analysis	False-positive NBS for metabolic disorders		463 children 9.5% (43) false-positive 92.7% (420) normal NBS
Kerruish et al. (2007) New Zealand [16]	Assess maternal anxiety, depressive symptoms, and perceptions of infant vulnerability related to NBS for susceptibility to T1D	Longitudinal quantitative Standardized assessments Researcher designed questionnaire	Increased risk for T1D	187 mothers	187 infants 20.3% (38) increased risk 39% (73) low risk 40.6% (76) not tested
Kerruish (2011) New Zealand [60]	Examine parents’ psychosocial reactions to NBS results that identified infants as having genetic susceptibility to T1D	Cross-sectional qualitative Semi-structured interviews	Increased risk for T1D	10 families 90% (9) mothers 1% (1) couple	10 infants
Kerruish (2016) New Zealand [61]	Assess long-term psychosocial effects of genomic NBS for susceptibility to T1D	Cross-sectional qualitative Semi-structured interviews	Increased risk for T1D	15 mothers	15 children
Kerruish et al. (2017) New Zealand [62]	Examine longer-term psychosocial effects of identifying genetic risk for T1D through NBS	Longitudinal quantitative Researcher designed questionnaire Standardized assessments	Increased risk for T1D	98 parent–child dyads 65.3% (64) low risk 34.7% (34) higher risk	
Kladny et al. (2011) USA [63]	Assess impact of genetic counseling on lives of families in which infants were identified as carrier of SCD gene identified through NBS	Cross-sectional intervention Non-randomized Researcher designed questionnaire	SCD carriers	114 parents	114 children
La Pean et al. (2012) USA [64]	Assess parents’ opinions about follow-up telephone call regarding NBS that identified infants as carriers for SCD or CF	Cross-sectional qualitative Semi-structured interviews	CF carriers SCD carriers	195 parents	195 infants 33% (65) CF carriers 66% (130) SC carriers
Lagoe et al. (2005) USA [65]	Compare impact of genetic counseling on parent uptake of genetic testing	Longitudinal intervention RCT Researcher designed questionnaire	CF carriers	61 parents of 31 infants 51% (31) mothers 49% (30) fathers	31 infants
Lang et al. (2009) USA [66]	Assess mothers’ understanding of NBS for SCD and CF and their knowledge of the genetics, symptoms, and treatments for each condition	Cross-sectional quantitative Researcher designed questionnaire	CF SCD	388 postpartum women 8.8% (34) SC carriers 0.26% (1) SCD 0.26% (1) CF carrier	
Lang et al. (2011) USA [67]	Assess parents’ knowledge of and attitudes about false-positive NBS for CF	Cross-sectional quantitative Researcher designed questionnaire	CF	90 parents	90 children
Lewis et al. (2006) Australia [68]	Investigate parental attitudes about CF carrier detection through NBS	Cross-sectional quantitative Researcher designed questionnaire	CF carriers	66 parents 45.5% (30) from 1996–1997 54.5% (36) from 2001	
Lipstein et al. (2009) USA [69]	Examine association between false-positive NBS for metabolic disorders and health care utilization	Cross-sectional quantitative Researcher designed questionnaire Standardized assessments	False-positive NBS metabolic disorders	337 mothers 59.3% (200) false-positive 40.7% (137) normal NBS	337 infants 59.3% (200) false-positive 40.7% (137) normal NBS
Locock and Kai (2008) England [70]	Explore parents’ experiences of and attitudes towards NBS screening for hemoglobin disorders	Cross-sectional qualitative Descriptive Semi-structured interviews	NBS for hemoglobin disorders	39 parents	
Miller et al. (2010) Canada [71]	Assess parents’ attitudes about NBS procedures that identify their infants as carrier of SCD genes	Cross-sectional qualitative Descriptive Semi-structured interviews Focus groups	SCD carriers	56 interviews 75% (42) providers 14.3% (8) advocates 10.7% (6) parents 12 focus groups with 66 participants 77.3% (51) new parents 22.7% (15) SCD lay consumers (parents or patients)	
Moran et al. (2007) England [72]	Assess the psychological impact of false-positive NBS on parents	Cross-sectional qualitative Descriptive Semi-structured interviews	False-positive NBS for CF	21 mothers	21 infants
Morrison and Clayton (2011) USA [73]	Assess the impact of receiving abnormal NBS results for metabolic or endocrine disorders on families	Cross-sectional mixed methods Structured interviews Standardized assessments	Abnormal NBS for metabolic/endocrine disorders	60 parents	60 infants
Newcomb et al. (2013) USA [74]	Assess mothers’ knowledge about NBS and attitudes about state retention of dried blood spots (DBS) for research	Cross-sectional quantitative Researcher designed questionnaire	NBS in general DBS retention	548 mothers of healthy infants	
Nicholls and Southern (2012) England [75]	Investigate how parents’ sources of information relate to their NBS experience	Cross-sectional mixed methods Researcher designed questionnaire Semi-structured interviews	NBS in general	172 parents 10.5% (18) interviewed 89.5% (154) questionnaires	
Nicholls and Southern (2013) England [76]	Examine factors that influence parental decision-making regarding NBS	Cross-sectional qualitative Descriptive Semi-structured interviews	NBS in general	18 parents	18 children under 2 years
O’Connor et al. (2018) Canada [77]	Examine impact of expanded NBS for metabolic disorders and CF on maternal psychosocial functioning and parenting stress	Cross-sectional quantitative Standardized assessments	Metabolic disorders CF	57 mothers 54.3% (31) true negative 14% (8) true positive 31.6% (18) false-positive	
Parsons et al. (2002) Wales [78]	Evaluate psychosocial implications of NBS for DMD	Cross-sectional mixed methods Semi-structured interviews Researcher designed questionnaire Standardized assessments	DMD	97 families 20.6% (20) positive NBS 18.6% (18) transient abnormality 16.5% (16) clinically dx 44.3% (43) without DMD	97 males ≥ 4 years
Parsons et al. (2003) Wales [79]	Examine psychosocial implications of identifying CF carriers through NBS	Cross-sectional mixed methods Semi-structured interviews Researcher designed questionnaire Standardized assessments	CF carriers	19 families of CF or CF carrier infants 47.4% (9) CF 52.6% (10) CF carriers 82 mothers from general population	
Perobelli et al. (2009) Italy [80]	Assess parents’ perspectives about diagnostic results for CF following positive NBS	Cross-sectional quantitative Researcher designed questionnaire	CF	33 families 33.3% (11) ambiguous 33.3% (11) CF 33.3% (11) negative NBS	33 children
Quigley et al. (2018) Ireland [81]	Evaluate intervention designed to increase parents’ knowledge of CF, reduce stress, and examine psychosocial effects of false-positive CF NBS results	Cross-sectional intervention RCT Researcher designed questionnaire Standardized assessments	False-positive NBS for CF	32 parents false-positive NBS 50% (16) intervention group 50% (16) control group	
Rueegg et al. (2016) Switzerland [82]	Assess parents’ satisfaction with NBS for CF	Cross-sectional quantitative Researcher designed questionnaire	CF	138 parents of 138 infants with positive NBS for CF	138 infants 64.5% (89) false-positive 34.1% (47) CF 1.4% (2) CFSPID
Salm et al. (2012) USA [83]	Examine parents’ perspectives about how best to communicate positive NBS results	Cross-sectional qualitative Descriptive Semi-structured interviews	CH CF CF carriers	203 parents of 106 infants with positive NBS 52.2% (106) mothers 47.8% (97) fathers	106 infants 34.9% (37) CH 24.5% (26) CF 40.6% (43) CF carriers
Sawyer and Glazner (2004) Australia [84]	Evaluate a 5-day residential assessment and education program for parents of infants with CF identified through NBS	Cross-sectional intervention Non-randomized Researcher designed questionnaire	CF	15 families 100% (15) mothers 80% (12) fathers	15 infants with CF
Sawyer et al. (2006) Australia [85]	Compare parents’ attitudes about reproductive technologies with their later reproductive behavior within context of NBS	Longitudinal quantitative Researcher designed questionnaire	CF	56 mothers 100% (56) baseline questionnaire 76.8% (43) follow-up interview at 5 years	56 children with CF
Schmidt et al. (2012) USA [86]	Describe parents’ experiences of false-positive NBS results	Cross-sectional qualitative Descriptive Semi-structured interviews Focus groups	False-positive NBS for multiple conditions	27 parents total 16 interviews with 17 parents 14 mothers 1 couple 1 father 2 focus groups with 10 parents 6 mothers 2 couples	16 infants 6–16 months old 14 false-positive NBS 2 controls
Scotet et al. (2000) France [87]	Assess 10 years of NBS for CF in France and impact on prenatal diagnosis in subsequent pregnancies	Cross-sectional quantitative Database Analysis	CF	Children had NBS for CF	343,756 total infants 112 CF NBS 6 CF clinical dx
Skinner et al. (2011) USA [88]	Document parental consent for NBS for FXS	Cross-sectional mixed methods Researcher designed questionnaire	FXS	1930 mothers 71.6% (1381) acceptors 28.4% (549) decliners	
Tarini et al. (2011) USA [10]	Examine health care use among infants with false-positive NBS results compared to infants with normal NBS results	Cross-sectional quantitative Database analysis	False-positive NBS results for endocrine or metabolic conditions		49,959 infants 49,141 normal NBS 818 false-positive (67.2% [550] endocrine; 32.3% [268] metabolic)
Temme et al. (2015) USA [89]	Evaluate effectiveness of communication intervention on parents’ knowledge about CF genetics and their child’s carrier status	Longitudinal intervention RCT Researcher designed questionnaire	CF carriers	96 parents of 100 CF carrier infants 58.2% (56) mothers 41.7% (40) fathers	100 CF carriers
Timmermans and Buchbinder (2010) USA [90]	Examine the social significance of uncertain NBS results	Cross-sectional qualitative Descriptive Semi-structured interviews	Ambiguous NBS for metabolic disorders	55 families	
Tluczek et al. (2005) USA [14]	Examine psychosocial risks associated with NBS for CF	Cross-sectional mixed methods Semi-structured interviews Standardized assessments	CF	32 parents 43.8% (14) CF-NBS 56.2% (18) healthy	32 infants 43.8% (14) CF-NBS 56.2% (18) healthy
Tluczek et al. (2006) USA [91]	Understand parents’ perceptions about genetic counseling received while awaiting their infant’s sweat test results following positive NBS for CF	Cross-sectional qualitative Grounded theory Semi-structured interviews	Positive NBS for CF	33 families 100% (33) mothers 94% (31) fathers	33 infants
Tluczek et al. (2009) USA [92]	Learn how parents were informed about NBS and how to improve parent education about NBS	Cross-sectional qualitative Descriptive Semi-structured interviews	CF diagnosis CF carriers CH	193 biological parents of 100 infants 93 couples 7 mothers	100 infants 16% (16) CF 34% (34) CF carriers 23% (23) CH 27% (27) healthy
Tluczek, Chevalier-McKechnie, et al. (2010) USA [93]	Examine psychosocial consequences of ambiguous NBS results for CF	Cross-sectional qualitative Grounded dimensional analysis Semi-structured interviews	Ambiguous NBS for CF	5 couples	5 infants
Tluczek, Clark, et al. (2010) USA [94]	Examine effects of NBS and neonatal diagnosis on quality of mother–infant interactions in the context of feeding	Cross-sectional mixed methods Standardized assessments Videotaped interactions	CF	130 mothers	130 infants 13.1% (17) CF 26.9% (35) CH 30.8% (40) CF carriers 29.2% (38) healthy
Tluczek, Orland, et al. (2011) USA [95]	Understand parents’ perspectives about false-positive NBS for CF	Cross-sectional mixed methods Semi-structured interview	False-positive results for CF	87 parents of 44 infants 50.6% (44) mothers 49.4% (43) fathers	44 infants
Tluczek, Becker, et al. (2011) USA [96]	Examine long-term health and health-related quality of life in patients diagnosed with CF through NBS compared to those diagnosed clinically	Cross-sectional quantitative Standardized assessments	CF		95 patients 47.4% (45) NBS 52.6% (50) clinical dx
Tluczek, Chevalier McKechnie, et al. (2011) USA [97]	Compare parent perceptions of child vulnerability as a function of diagnostic severity following NBS	Cross-sectional quantitative Researcher designed questionnaire Standardized assessments	CF carriers CF CH	257 parents of 136 infants 52.9% (136) mothers 47.1% (121) fathers	136 infants 16.9% (23) CF 26.5% (36) CH 29.4% (40) CF carriers 27.2% (37) normal NBS
Tluczek et al. (2014) USA [98]	Examine psychological functioning of youths identified with CF through NBS	Cross-sectional quantitative Standardized assessments	CF	72 parents of 81 youths 88.9% (64) mothers 11.1% (8) fathers	81 youths 16% (13) CF-NBS 32.1% (26) CF-clinical dx 51.9% (42) healthy
Tluczek et al. (2015) USA [99]	Examine factors that mediate parent–infant relationships 12 months after positive NBS for CF or CH	Cross-sectional mixed methods Standardized assessments Videotaped interactions	CF CF carriers CH	249 parents of 131 infants 52.6% (131) mothers 47.4% (118) fathers	131 infants 17.6% (23) CF 26.7% (35) CH 29% (38) CF carrier 26.7% (35) NS
Tluczek et al. (2019) USA [100]	Examine factors affecting parenting, parents’ perceptions of their children’s vulnerability, and protectiveness following intermediate CF diagnosis	Cross-sectional mixed methods Researcher designed questionnaire Standardized assessments	Intermediate CF CF	110 parents 36.4% (40) CF 18.2% (20) intermediate dx 45.5% (50) normal NBS	110 children 36.4% (40) CF 18.2% (20) intermediate dx 45.5% (50) normal NBS
Tu et al. (2012) China [101]	Assess impact of NBS for metabolic disorders on parental stress, perceptions of the child’s health, and family relationships	Cross-sectional quantitative Researcher designed questionnaire Standardized assessments	False-positive NBS for metabolic disorders	Parents of 91 infants 88 mothers 53.4% (47) false-positive 46.6% (41) normal NBS 41 fathers 56.1% (23) false-positive 43.9% (18) normal NBS	91 infants 53.8% (49) false-positive NBS 46.2% (42) normal NBS
Ulph et al. (2011) England [102]	Examine parents’ prior knowledge, service use, screening decisions, and communication with family members following identification of hemoglobin disorders through NBS	Cross-sectional qualitative Descriptive Semi-structured interviews	Carriers for hemoglobin disorders	37 parents 75.7% (28) mothers 24.3% (9) fathers	
Ulph et al. (2014) England [103]	Examine parents’ intentions to inform their child of NBS carrier result	Cross-sectional qualitative Descriptive Semi-structured interviews	CF carriers SCD carriers	67 family members 73.1% (49) mothers 3% (2) grandparents 23.9% (16) fathers	51 infants
Ulph et al. (2015) England [104]	Examine effects of informing parents that their child is a carrier of CF or SCD identified through NBS	Cross-sectional qualitative Descriptive Semi-structured interviews	CF carriers SCD carriers	67 family members 73.1% (49) mothers 3% (2) grandparents 23.9% (16) fathers	51 infants 52.9% (27) CF carriers 47.1% (24) SCD
Van Der Sluijs Veer et al. (2008) Netherlands [105]	Examine long-term health-related quality of life and developmental milestones of children diagnosed with CH through NBS	Cross-sectional quantitative Researcher designed questionnaire	CH		270 adults 25.6% (69) CH 74.4% (201) healthy
Vernooij-van Langen et al. (2014) Netherlands [106]	Evaluate effectiveness of parent education in reducing stress and anxiety related to NBS for CF	Cross-sectional intervention Non-randomized Researcher designed questionnaire	False-positive NBS results for CF	208 parents 29.8% (62) false-positive 70.2% (146) normal NBS	208 infants 29.8% (62) false-positive 70.2% (146) normal NBS
Waisbren et al. (2003) US [107]	Assess impact of false-positive NBS for metabolic genetic disorders on families	Cross-sectional quantitative Researcher designed questionnaire Standardized assessments	Metabolic disorders	407 parents of 258 infants 62.4% (254) mothers 37.6% (153) fathers	258 infants 19.4% (50) NBS 12.8% (33) clinical dx 36% (94) false-positive 31.4% (81) healthy
Waisbren et al. (2004) US [108]	Compare parenting stress following infants’ diagnosis of biochemical genetic disorders through NBS vs. clinically	Cross-sectional quantitative Researcher designed questionnaire Standardized assessments	Biochemical disorders	262 parents of 263 children 89% (234) mothers 10% (26) fathers 1% (2) grandparents	263 children with biochemical disorders 52.9% (139) NBS 47.1% (124) clinical dx
Wheeler et al. (2001) US [109]	Evaluate outcomes of genetic counseling for parents of infants with positive NBS for CF	Cross-sectional quantitative	Positive NBS for CF	101 families of 102 newborns 2 couples–nonbiological parents 99 couples–biological parents 4% (4) couples tested prenatally 10.1% (10) couples declined testing 14.1% (14) couples one parent tested 71.7% (71) couples both parents tested	102 newborns 2% (2) CF 98% (100) false-positive

Key: congenital hypothyroid (CH), cystic fibrosis (CF), cystic fibrosis screen positive inconclusive diagnosis (CFSPID), Duchenne muscular dystrophy (DMD), diagnosis (dx), fragile X syndrome (FXS), newborn screening (NBS), sickle cell disease (SCD), type 1 diabetes (T1D).

**Table 4:** Recommendations.

**Improve parents’ general knowledge of NBS** Educate parents about NBS during prenatal health visits in the third trimester as well as at the time of NBS blood specimen collection. Include fathers in NBS education. Explain the difference between a screening and diagnostic test. Explain that if a result is positive, the child will require additional testing to confirm or rule out the condition. Use multiple communication modes in parents’ preferred language (i.e., verbal, written, and educational videos). Consider implementing techniques designed to demonstrate understanding (i.e., use of teach-back).
**Improve parents’ understanding of NBS positive results and next steps** Avoid contacting parents when they will likely have difficulty obtaining answers to their questions from health care providers (i.e., Friday or just before a holiday). Limit wait time for follow-up diagnostic testing to no more than three days. Develop NBS protocols that ensure results will be communicated by health professionals who are knowledgeable about NBS, as well as about the condition for which the child screened positive, can accurately interpret the test results, and can explain the implications. If possible, communicate results to both parents in person. Recognize that parents whose primary languages are other than the national language in the region where the NBS program is conducted are at high risk for misunderstanding. Use interpreters when needed to ensure parents understand the NBS results and next steps. Explain the meaning of “positive” vs. “negative” NBS results. The meanings of these terms in the context of NBS are counterintuitive to lay parlance (i.e., negative NBS results are good news because no health conditions were found while positive results suggest the possible presence of a health condition). Explain what parents can anticipate during follow-up tests, and how they can prepare. Encourage both parents to attend appointments so they receive the same information simultaneously. Create parent-friendly centralized systems for scheduling appointments with specialists. If parents have other children, suggest they find an adult to stay at home with the children while the parents attend the appointment, or bring an additional adult to the clinic to care for the other children so parents can focus on the information provided. Offer parents realistic reassurance and instill a sense of hopefulness about the child’s future, as appropriate.
**Reduce parents’ emotional distress and ensure their understanding of diagnostic test results and related implications** Choose a clinic space for family counseling that is quiet, private, and has minimal distractions or interruptions. Begin the session by congratulating parents on the birth of the child. Recognize that parents’ psychological responses to positive NBS results can vary widely depending on their prior knowledge. Be careful not to assume anything about parents’ psychological reactions. Assess each parent’s emotional state, pre-existing knowledge (including genetics), ways of coping with uncertainty, and need for information. Inquire about the infant’s health. Use insights gleaned from this discussion to further assess parents’ psychological mindset and individualize information shared on the family’s particular circumstances. Identify parents’ most pressing concerns, express empathy, and address their concerns first. Tailor content, timing, and pace of information delivery based on parents’ preferences and understanding. Use checklists and sequence information in order of importance. Explain the meaning of false-positive results and any related implications. Create a reciprocal dialogue in which providers follow parents’ lead in conversation. Encourage parents to ask questions and share their thoughts/feeling. Use simple language, avoid jargon, and explain esoteric medical terms. Apply multiple communication modes, especially for complex concepts (i.e., verbal explanations, diagrams that illustrate genetic transmission, brochures that summarize content, and educational videos—all in the parent’s preferred language). Use teach-back methods to ensure parents’ accurate understanding. If parents misunderstand information provided, frame the error as a function of the provider’s failure to clearly communicate, not parents’ shortcomings. Direct parents to credible online resources. Offer parents realistic reassurance and hope about their child’s health and future. Provide parents opportunities to recontact providers if they have additional questions or concerns after the session. Send parents a follow-up letter, in their preferred language, that summarizes the session, test results, and next steps if relevant.
**Support families beyond the NBS process** Offer parents guidance in communicating genetic information with extended family members. As children mature, assist parents in identifying optimal timing and developmentally applicable ways to inform children about genetic findings and implications for the child’s future. Observe for signs of continued parental anxiety, unfounded perceptions of child vulnerability, or over utilization of health care services. If overprotectiveness arises, revisit parents’ understanding of NBS findings and correct misconceptions. Encourage developmentally appropriate child-rearing practices while respecting parents’ cultural norms. Refer the family for additional mental health assessment as needed.
